# Error in Conflict of Interest and Funding/Support

**DOI:** 10.1001/jamanetworkopen.2025.51007

**Published:** 2025-12-04

In the Invited Commentary titled “Adolescent Vulnerability to Consumer Chatbots—Artificial Agents and Genuine Risk,”^1^ published October 23, 2025, the Conflict of Interest Disclosures, Funding/Support, and Role of the Funder sections were omitted from the article information. This article has been corrected.^1^
